# Supplementary material for: Impact of Mygalin on Inflammatory Response Induced by Toll-like Receptor 2 Agonists and IFN-γ Activation
Source: Int J Mol Sci. 2024 Sep 30;25(19):10555. doi: 10.3390/ijms251910555 (PMC11476598; doi:10.3390/ijms251910555)
Supplement: Supplementary file 1 [file ijms-25-10555-s001.zip › ijms-3214917-supplementary.pdf]

Supplementary information.

**Table S1.** Molecular interactions between ligand and TLR2/1

| Pam3CSK4 | TLR2 residue | Interaction type |
|---|---|---|
| K4 | 294N | Ionic |
| S2 | 324L | H bond |
| Chain 2 | 325F | H bond |
| S2 | 327D | H bond |
| K3 | 327D | H bond |
| Chain 2 | 347K | H bond |
| Chain 2 | 349F | H bond |
| Chain 2 | 350L | H bond |
| Chain 1 | 266L | Hydroph. |
| Chain 1 | 270M | Hydroph. |
| Chain 1 | 282L | Hydroph. |
| Chain 2 | 282L | Hydroph. |
| Chain 2 | 284F | Hydroph. |
| Chain 1 | 295F | Hydroph. |
| Chain 2 | 306P | Hydroph. |
| Chain 1 | 309V | Hydroph. |
| Chain 2 | 312L | Hydroph. |
| Chain 1 | 312L | Hydroph. |
| Chain 1 | 314I | Hydroph. |
| Chain 1 | 317L | Hydroph. |
| Chain 2 | 319I | Hydroph. |
| Chain 2 | 325F | Hydroph. |
| K4 | 326Y | Hydroph. |
| Chain 1 | 328L | Hydroph. |
| Chain 1 | 334L | Hydroph. |
| Chain 1 | 338L | Hydroph. |
| Chain 1 | 341I | Hydroph. |
| Chain 1 | 343V | Hydroph. |
| Chain 2 | 346S | Hydroph. |
| Chain 2 | 348V | Hydroph. |
| Chain 2 | 349F | Hydroph. |
| Chain 1 | 351V | Hydroph. |
| Chain 1 | 352P | Hydroph. |
| Chain 2 | 352P | Hydroph. |
| Chain 1 | 355L | Hydroph. |
| Chain 2 | 376Y | Hydroph. |

| Mygalin | TLR2 residue | Interaction type |
|---|---|---|
| Ring 2 | 322F | H bond |
| Ring 1 | 324L | H bond |
| Spermidine | 325F | H bond |
| Ring 2 | 327D | H bond |
| Ring 1 | 347K | H bond |
| Spermidine | 350L | H bond |
| Spermidine | 325F | Hydrop |
| Ring 1 | 326Y | Hydrop |
| Ring 2 | 346S | Hydrop |
| Ring 1 | 347K | Hydrop |
| Ring 1 | 348V | Hydrop |
| Ring 1 | 349F | Hydrop |
| Spermidine | 350L | Hydrop |
| Spermidine | 352P | Hydrop |

| Mygalin | TLR1 | Interaction |
|---|---|---|
| Spermidine | 314F | H bond |
| Spermidine | 316Q | H bond |
| Spermidine | 312F | Hydrop |
| Ring 2 | 313G | Hydrop |
| Ring 2 | 315P | Hydrop |
| Spermidine | 316Q | Hydrop |

| Pam3CSK4 | TLR6 residue | Interaction type |
|---|---|---|
| Chain 3 | 312F | H bond |
| Chain 3 | 313G | H bond |
| Chain 3 | 316Q | H bond |
| K5 | 318Y | H bond |
| Chain 3 | 320Y | H bond |
| Chain 3 | 258W | Hydroph. |
| Chain 3 | 307V | Hydroph. |
| Chain 3 | 311V | Hydroph. |
| Chain 3 | 312F | Hydroph. |
| K5 | 315P | Hydroph. |

| | | |
|---|---|---|
| Chain 3 | 319I | Hydroph. |
| Chain 3 | 323F | Hydroph. |
| Chain 3 | 333V | Hydroph. |
| Chain 3 | 337R | Hydroph. |
| Chain 3 | 338M | Hydroph. |

**Table S2**. Molecular interactions between ligands and TLR2/6

| Pam2CSK4 | TLR2 residue | Interaction |
|---|---|---|
| K2 | D294 | Ionic |
| Chain1 | F349 | Hbond |
| Chain1 | L350 | Hbond |
| Chain2 | P352 | Hbond |
| K2 | Y326 | Hbond |
| S2 | D327 | Hbond, |
| Chain2 | F256 | Hydroph. |
| Chain1 | L261 | Hydroph. |
| Chain2 | F266 | Hydroph. |
| Chain2 | L269 | Hydroph. |
| Chain2 | L270 | Hydroph. |
| Chain2 | V282 | Hydroph. |
| Chain1 | F284 | Hydroph. |
| Chain2 | F284 | Hydroph. |
| Chain1 | 295F | Hydroph. |
| Chain2 | L306 | Hydroph. |
| Chain2 | V312 | Hydroph. |
| Chain1 | I314 | Hydroph. |
| Chain2 | L317 | Hydroph. |
| Chain1 | I319 | Hydroph. |
| Chain1 | F325 | Hydroph. |
| K2 | Y326 | Hydroph. |
| K1 | D327 | Hydroph. |
| Chain2 | F328 | Hydroph. |
| Chain2 | L335 | Hydroph. |
| Chain2 | I341 | Hydroph. |
| Chain2 | V343 | Hydroph. |
| Chain1 | K347 | Hydroph. |
| Chain1 | V348 | Hydroph. |
| Chain1 | F349 | Hydroph. |
| Chain1 | V351 | Hydroph. |
| Chain2 | P352 | Hydroph. |
| C1 | P352 | Hydroph. |
| Chain2 | F355 | Hydroph. |

| Pam2CSK4 | TLR6 | Interaction |
|---|---|---|
| K1 | L318 | Ionic |
| C1 | 317F | H bond |
| K1 | L318 | H. bond |
| K1 | L318 | Hydroph. |
| C1 | F319 | H bond |
| C1 | F319 | Hydroph. |
| C1 | K321 | H bond |

| Zymosan monosaccharid | TLR2 residue | Interaction Type |
|---|---|---|
| 5 | 322F | Hbond |
| 5 | 323Y | Hbond |
| 6 | 324L | Hbond |
| 5 | 327D | Hbond |
| 3 | 328L | Hbond |
| 3 | 343V | Hbond |
| 4 | 346S | Hbond |
| 4 | 350L | Hbond |
| 1 | 261L | Hydroph |
| 1 | 266F | Hydroph |
| 1 | 284F | Hydroph |
| 3 | 289L | Hydroph |
| 3 | 295F | Hydroph |
| 2 | 312V | Hydroph |
| 3 | 314I | Hydroph |
| 3 | 317L | Hydroph |
| 4 | 325F | Hydroph |
| 6 | 326Y | Hydroph |
| 2 | 335L | Hydroph |
| 3 | 343V | Hydroph |
| 4 | 347K | Hydroph |
| 4 | 348V | Hydroph |
| 4 | 349F | Hydroph |
| 5 | 352P | Hydroph |

| Zymosan | TLR6 | Interaction |
|---|---|---|
| 7 | 292I | Hbond |
| 7 | 293D | Hbond |
| 5 | 318L | Hydroph |
| 6, 7 | 319F | Hydroph |
| 6 | 320S | Hydroph |
| 5, 6 | 321K | Hydroph |

| Mygalin | TLR2 residue | Interaction |
|---|---|---|
| Ring 2 | 327D | HBond |
| Spemidine | 326Y | HBond |
| Spemidine | 350L | HBond |
| Spemidine | 325F | Hydroph. |
| Ring 1 | 326Y | Hydroph. |
| Ring 2 | 343V | Hydroph. |
| Ring 2 | 351V | Hydroph. |
| Ring 2 | 355F | Hydroph. |
| Ring2 | 328L | Hydroph. |
| Spemidine | 348V | Hydroph. |
| Spemidine | 349F | Hydroph. |
| Spemidine | 350L | Hydroph. |
| Spemidine | 352P | Hydroph. |

| Mygalin | TLR6 | Interaction |
|---|---|---|
| Ring 1 | 319F | HBond |
| Ring 1 | 318L | Hydroph. |
| Spemidine | 321K | Hydroph. |

| | | |
|---|---|---|
| C1 | K321 | Hydroph. |
